# Early hippocampal synaptic plasticity and episodic like-memory deficits in a transgenic mouse model of Alzheimer disease - involvment of corticosterone

**DOI:** 10.1186/1750-1326-8-S1-P62

**Published:** 2013-10-04

**Authors:** Fabien Lanté, Magda Chafai, Elisabeth Raymond, Ana Rita Salgueiro Peirera, Ingrid Bethus, Helene Marie

**Affiliations:** 1 Institut de Pharmacologie Moléculaire et Cellulaire (IPMC), Vablonne, France; 2 Present address: Institut des Neurosciences, Grenoble, France

## Background

The etiology of Alzheimer's disease (AD) is unclear and no cure is yet available. The function of the hippocampus, a key structure responsible for memory encoding and consolidation, is affected early in AD leading to progressive irreversible memory loss. There is strong evidence that AD onset is, at least partly, due to accumulation within the hippocampus of peptides processed from the amyloid precursor protein APP, such as amyloid-beta (A?). Also, several studies demonstrated an abnormal elevation in the main stress hormone, cortisol (CORT in mice), in the initial phase of AD in patients and in mouse models. Here, we investigated if early co-accumulation of CORT and APP-derived peptides could be a main trigger driving the onset of memory deficits in AD.

## Material and methods

We used the Tg2576 mouse model, which accumulates hippocampal A? with age and displays hippocampus-dependent memory loss. We first investigated the mechanisms underlying long term depression (LTD) and long term potentiation (LTP) in CA1 region of hippocampal slices of Tg2576 mice. We also evaluated circadian plasma CORT levels by ELISA. Finally, episodic memory processing, the type of memory first affected in AD patients, and the involvement of CORT signaling were investigated, using an elaborated version of the object recognition (OR) task, which can probe for the "what, where and when" components of episodic memory.

## Results

We show that at 4 months of age, an age where A? begins to accumulate, Tg2576 mice already display enhanced NMDA receptor-dependent LTD, but unchanged LTP, in CA1 pyramidal neurons compared to WT littermates. We further show that while morning CORT levels are identical in 4 months old WT and Tg2576 mice, the evening levels are significantly higher in Tg2576 mice. These data suggest alterations in CORT signaling. We further demonstrate that the LTD alteration observed in these Tg2576 mice is prevented by 4 days of subcutaneous injection of the glucocorticoid receptor antagonist, RU486. Finally, our data show that WT mice have a good episodic-like memory. By contrast, 4 months old Tg2576 mice show impairments in the spatial component "where" of this episodic-like memory. However, this episodic memory deficit can be prevented by the RU486 treatment.

## Conclusions

These data identify CORT signaling as a factor in the early occurrence of hippocampal dysfunction with concomitant episodic memory deficits in this AD mouse model.

